# Author Correction: Ultrafast photonic reinforcement learning based on laser chaos

**DOI:** 10.1038/s41598-018-23476-2

**Published:** 2018-04-05

**Authors:** Makoto Naruse, Yuta Terashima, Atsushi Uchida, Song-Ju Kim

**Affiliations:** 1 Strategic Planning Department, National Institute of Information and Communications Technology, 4-2-1 Nukui-kita, Koganei, Tokyo 184-8795 Japan; 2 Department of Information and Computer Sciences, Saitama University, 255 Shimo-Okubo, Sakura-ku, Saitama, Saitama, 338-8570 Japan; 3 Graduate School of Media and Governance, Keio University, 5322 Endo, Fujisawa, Kanagawa 252-0882 Japan

Correction to: *Scientific Reports* 10.1038/s41598-017-08585-8, published online 18 August 2017

This article contains an error in the Acknowledgements section.

“This work was supported in part by the Core-to-Core Program A. Advanced Research Networks and the Grants-in-Aid for Scientific Research (A) from the Japan Society for the Promotion of Science.”

should read:

“This work was supported in part by the Core-to-Core Program A. Advanced Research Networks and the Grants-in-Aid for Scientific Research (A) (JP17H01277) and (B) (JP16H03878) from the Japan Society for the Promotion of Science.”

